# QTL Mapping and Identification of Candidate Genes for Heat Tolerance at the Flowering Stage in Rice

**DOI:** 10.3389/fgene.2020.621871

**Published:** 2021-01-22

**Authors:** Lei Chen, Qiang Wang, Maoyan Tang, Xiaoli Zhang, Yinghua Pan, Xinghai Yang, Guoqing Gao, Ronghua Lv, Wei Tao, Ligeng Jiang, Tianfeng Liang

**Affiliations:** ^1^Key Laboratory of Crop Cultivation and Farming System, College of Agriculture, Guangxi University, Nanning, China; ^2^Guangxi Academy of Agricultural Sciences/Guangxi Key Laboratory of Rice Genetics and Breeding, Rice Research Institute, Nanning, China

**Keywords:** rice, flowering stage, heat tolerance, QTL, BSA-seq, candidate gene

## Abstract

High-temperature stress can cause serious abiotic damage that limits the yield and quality of rice. Heat tolerance (HT) during the flowering stage of rice is a key trait that can guarantee a high and stable yield under heat stress. HT is a complex trait that is regulated by multiple quantitative trait loci (QTLs); however, few underlying genes have been fine mapped and cloned. In this study, the F_2:3_ population derived from a cross between Huanghuazhan (HHZ), a heat-tolerant cultivar, and 9311, a heat-sensitive variety, was used to map HT QTLs during the flowering stage in rice. A new major QTL, *qHTT8*, controlling HT was identified on chromosome 8 using the bulked-segregant analysis (BSA)-seq method. The QTL *qHTT8* was mapped into the 3,555,000–4,520,000 bp, which had a size of 0.965 Mb. The candidate region of *qHTT8* on chromosome 8 contained 65 predicted genes, and 10 putative predicted genes were found to be associated with abiotic stress tolerance. Furthermore, qRT-PCR was performed to analyze the differential expression of these 10 genes between HHZ and 9311 under high temperature conditions. *LOC_Os08g07010* and *LOC_Os08g07440* were highly induced in HHZ compared with 9311 under heat stress. Orthologous genes of *LOC_Os08g07010* and *LOC_Os08g07440* in plants played a role in abiotic stress, suggesting that they may be the candidate genes of *qHTT8*. Generally, the results of this study will prove useful for future efforts to clone *qHTT8* and breed heat-tolerant varieties of rice using marker-assisted selection.

## Introduction

Rice (*Oryza sativa* L.) is a major staple food crop for nearly half of the world's population (Pan et al., 2020). As global temperatures have increased in recent years, extreme, high temperatures have led to serious losses in yield, decreases in grain quality and reductions in harvest index, especially during the flowering stage, which has a net negative impact on the normal seed setting of rice (Jagadish et al., 2012). Average global temperatures are expected to increase by 2–3°C over the next 30–50 years (Hatfield and Prueger, 2015). However, rice yields are expected to decrease by 10% for every increase in daily maximum and minimum temperature of 1°C (Welch et al., 2010). In addition, the average daily temperature is expected to exceed 35°C for several consecutive days, which will lead to spikelet sterility and abnormal pollination, seriously reducing the seed-setting rate (IPCC, 2007). Current strategies to deal with high-temperature stress via alterations to technical and management systems are insufficient for sustaining yields (Driedonks et al., 2016). There is thus an urgent need to breed heat-tolerant rice varieties.

Many researchers claimed that the most sensitive growth stage of rice to heat stress was the flowering time (Baliuag et al., 2015; Nubankoh et al., 2020). And the study of HT at the flowering stage has become a major focus of rice breeding. Understanding the genetic mechanisms of HT and developing heat-tolerant varieties are essential for the ability of rice to cope with future global warming (Ye et al., 2015a,b). Germplasm resources are the material basis for the breeding of new rice varieties. The most effective method is to select different types of heat-tolerant materials to identify different rice germplasm resources, characterize HT and build a robust population, which can provide a foundation for the breeding of stress-tolerant varieties, a reference for the identification of heat-tolerant genes and a means for the exploration of heat-tolerant mechanisms. Effective measures for dealing with high-temperature stress in rice include the identification of heat-tolerant genes, the acquisition of intermediate materials and the cultivation of heat-tolerant varieties (Kilasi et al., 2018).

Much research over the past decades has focused on the mining of heat-tolerant genes in rice, primarily through the construction of different genetic populations (Cao et al., 2020). Yield or quality traits related to HT have been used as the evaluation indexes for rice heat-tolerant QTL analysis (Cao et al., 2002; Zhu et al., 2006; Asako et al., 2007; Xiao et al., 2011; Ye et al., 2012, 2015a,b; Nubankoh et al., 2020). These previous works have resulted in the detection of heat-tolerant QTLs in different regions of multiple chromosomes. Recent work has focused on the physical and chemical properties and agronomic characters of rice during each sensitive period, and has resulted in several breakthroughs in the study of heat-tolerance mechanisms. Specifically, numerous achievements have been made in research on rice HT molecular genetics, including the mapping of several rice HT QTLs. However, few QTLs related to rice HT have been cloned (Cao et al., 2020).

Genetic analysis has revealed that HT at the flowering stage in rice is a complex quantitative trait controlled by multiple genes. The resistance of rice to high temperatures shows variety specificity, which indicates that genetic factors contribute the most to explaining variation in HT among rice varieties. With the development and wide application of molecular biology and genomic tools in recent years, there has been an increasing number of QTL-mapping studies of rice HT using molecular markers. QTLs/genes for rice HT have been mapped across all 12 chromosomes using different types of molecular markers, such as RFLPs, SSRs, and SNPs, which has facilitated the identification of chromosomal regions associated with tolerance of high temperatures (Supplementary Table 1). In addition, different parents and types of mapping populations (e.g., F_2_, F_2:3_ lines, BC, NILs, RILs, CSSLs, and DH) have been used to analyze QTLs/genes with different yields (e.g., seed setting rate, spikelet fertility, pollen fertility, grain weight, flowering time and heading days) and quality traits (e.g., white-back kernels, basal-white grain and gel consistency) related to rice heat-stress tolerance at different stages, such as the seeding and reproductive stages (Cao et al., 2002, 2020; Zhu et al., 2006, 2017; Asako et al., 2007; Tabata et al., 2007; Chen et al., 2008; Jagadish et al., 2008, 2010; Xiao et al., 2011; Cheng et al., 2012; Ye et al., 2012, 2015a,b; Murata et al., 2014; Tazib et al., 2015; Wada et al., 2015; Zhao et al., 2016; Shanmugavadivel et al., 2017; Nubankoh et al., 2020).

The presence of similar heat-tolerant QTLs in rice indicates that the heat-tolerant metabolic pathways might be conserved among different rice varieties and that some QTLs with greater effects could be stably expressed. However, some heat-tolerant QTLs have not been consistently detected, which may be related to the different genetic backgrounds of varieties, or differences in environmental conditions among tests. Rice HT is characterized by quantitative trait inheritance, and its molecular mechanism is relatively complex. An important line of research on the molecular mechanism of rice HT is the determination of genes involved in the regulation of the response of rice to heat stress. Although QTLs for rice HT at the flowering stage have been mapped on all 12 chromosomes using various rice populations, the additive effect of each QTL is relatively low. As a result, introducing one or a few QTLs into a variety may not sufficiently increase its HT (Ye et al., 2015a,b). Therefore, the fine mapping, validation and characterization of more major QTLs and the design of functional SNP chips with QTL-linked markers are necessary for accelerating the selection and incorporation of multiple QTLs and, in turn, improving the efficiency of rice heat-tolerant breeding.

Here, the heat-tolerant variety HHZ and the heat-sensitive variety 9311 studied by our research group in a previous study were hybridized (F_1_) and then continuously self-crossed to develop source materials (F_2_ and F_2:3_) for HT identification and QTL mapping (Cao et al., 2008; Wang et al., 2020). A total of 365 F_2:3_ populations were selected for HT evaluation at the flowering stage. The QTLs for spikelet fertility under high-temperature stress were rapidly identified using the BSA-seq method combined with whole-genome resequencing (WGS) technology (Takagi et al., 2013; Zou et al., 2016). Finally, one major QTL, *qHTT8*, controlling HT at the flowering stage was identified on chromosome 8. Furthermore, we performed qRT-PCR to study the expression of ten putative genes under heat stress. A phylogenic analysis suggested that *LOC_Os08g07010* and *LOC_Os08g07440* were the two candidate genes controlling HT at the flowering stage in rice. Generally, the results of this study will aid future efforts to improve the HT in rice.

## Materials and Methods

### Rice Materials

HHZ and 9311 are both conventional *indica* rice varieties that were kindly provided by the Guangdong Academy of Agricultural Sciences and Huazhong Agricultural University, respectively. In past decades, the F_2:3_ population was widely used for rapid QTL mapping around different crops because of its short construction time and obvious segregation of the allelic characteristics of the parent strains parental lines (Austin and Lee, 1995; Fahliani et al., 2010; Park et al., 2013). A set of 365 F_2:3_ lines derived from a cross between HHZ, a heat-tolerant cultivar (Cao et al., 2008; Wang et al., 2020), and 9311, a heat-susceptible cultivar from our previous work, was used to evaluate rice HT at the flowering stage in this study. In 2018, F_2:3_ lines and their parents were planted in the net-house of Guangxi Academy of Agricultural Sciences, Nanning, Guangxi, China.

### Evaluation of HT in F_2:3_ At the Flowering Stage

An F_2:3_ population of 365 individuals was planted in plastic pots under natural conditions until heading. At the start of heading, 3–5 uniform panicles with the opened florets carefully removed, were marked with PVC tags (Jagadish et al., 2007). The plants were then moved into a phytotron. During this period, the spikelets in the panicle were exposed to 38/24°C day/night temperatures with 6 h (from 09:30 am to 3:30 pm) of 38°C during the day (Liang et al., 2016). After 3 days of exposure to high temperature, the plants were moved back to the net house and were grown to maturity. After harvest, the labeled panicles were tested for seed set, and the numbers of fully filled grains (N_FG_), partially filled grains (N_PG_) and empty grains (N_EG_) were counted (Liang et al., 2016). Each spikelet was pressed between the thumb and forefinger to determine whether the grain was filled or not. Both partially and fully filled spikelets were categorized as filled spikelets (Mohammed and Tarpley, 2009; Rang et al., 2011). Next, the absolute spikelet fertility percentage was calculated using the formula below, and HT was assessed based on absolute fertility at high temperature (Jagadish et al., 2010; Cao et al., 2020).

Spikelet fertility (%) = (N_FG_ + N_PG_)/ (N_FG_ + N_PG_ + N_EG_) × 100.

### Whole Genome Re-Sequencing and BSA-seq Analysis

According to the phenotypic characterization of HT of derived F_2:3_ lines at anthesis under heat stress, pools of tolerant and sensitive bulk samples (*n* = 50, each group) were constructed from 365 F_2_ individuals, and the same amount of DNA of fresh leaves was extracted from each plant and was evenly mixed.

After the sample DNA was quantified by a Nanodrop 2000, the DNA in each mixing pool was equally mixed. The parents and the mixing pool DNA sequences were segmented into random fragments using ultrasound. The segmented DNA was successively repaired at the end; A was added at the 3′ end, and the sequencing connector was connected. Next, the magnetic beads were used to absorb and enrich the fragments with lengths of approximately 400 bp, which were amplified by PCR to form a sequencing library. After inspection of the constructed library, the qualified library was sequenced using the Illumina NovaSeq 6000 platform. The sequencing approach used was Illumina PE150, and the total sequencing read length was 300 bp. After the low-quality reads (raw data) were filtered out, the remaining reads (clean data) were aligned to the *Oryza sativa L. ssp. japonica cv*. Nipponbare reference genome using BWA software. The location of the sequence (i.e., the BAM file) was then obtained. The best practices pipeline in GATK software was used to correct BAM files and detect SNPs and small InDels.

SnpEff software and gene prediction information of the reference genome were used to annotate the variation function, and the function annotation information for SNPs and InDels was obtained. Based on the characteristics of the data for parents and mutation pools, the SNP-index (the SNP frequency) value was calculated for the BSA association analysis to locate the target loci. For the SNP and InDel loci among the samples obtained by filtering and screening, the SNP-index values of each locus in the heat-tolerant mixed pool (T-pool)/ the sensitive mixed pool (S-pool) were calculated. The average SNP-index values of all SNPs in the window were then counted as the SNP-index of that window. The window was sliding, with a 500 kb window size and a 5-kb increment. The SNP-index of the T-pool and S-pool was defined as the ratio between the HHZ SNP and the total number of reads corresponding to the SNP. The Δ (SNP-index) was calculated according to the formula Δ(SNP-index) = [(SNP-index of T-pool) – (SNP-index of S-pool)]. Because of the linkage between the heat-tolerant loci and surrounding markers, the SNP-index in the T-pool was closer to 1, whereas the SNP-index in the S-pool was closer to 0. Because of weak linkage or a lack of linkage, the loci were randomly distributed, and the SNP-index of the other normal loci was 0.5. A thousand replacement tests were performed, and the region with the most differences in SNP-index values between the two pools (Δ) (using the 99.9% confidence level as the threshold for screening) was the candidate region of the target trait correlation. All candidate genes were analyzed by GO enrichment analysis (Gene Ontology, http://www.geneontology.org/) based on a Fisher's exact test and a Yekutieli multitest adjustment using a 5% false-positive detection threshold (Yang et al., 2017).

### qRT-PCR Analysis of Candidate Genes in the Mapped Region

HHZ and 9311 were cultivated until the flowering stage, and 3 plants of each were subjected to 38°C heat stress from 9:30 am to 3:30 pm during the day for 3 days in a phytotron. Panicle tissues were sampled at different times from the initiation of heat stress, namely at 8:30 am, 10:30 am, 12:30 pm, 3:30 pm and 4:30 pm every day. Each sampling period was arranged in order from No. 1 to 15, among which, No. 5, 10, and 14 were not measured out of the convenience of analysis. Total RNA was extracted from panicles using the TRIzol® reagent (Invitrogen, Carlsbad, USA), and 2 μg of DNaseI-treated RNA was used as the template for cDNA synthesis using the PrimeScript^TM^ RT reagent Kit with gDNA Eraser. A Bio-Rad CFX96 Real-Time system (Bio-Rad Laboratories, Inc., USA) was used to perform quantitative real-time PCR in 10-μL mixtures: 5 μL of 2 × Green qPCR MasterMix, 1 μL of cDNA, 0. 5 μL of each primer (10 μM), and 3.5 μL of ddH_2_O. Amplification steps were 95°C for 30 s, 40 cycles of (95°C for 5 s, 60°C for 30 s), and 65°C for 5 s, 95°C for 15 s, 60°C for 30 s, 95°C for 15 s. Relative gene expression levels were calculated using the 2^−ΔΔCt^ method (Livak and Schmittgen, 2001). Ubiquitin (*UBQ*) was used as the internal control and at least three replicates were performed for each experiment. Primers used for qRT-PCR are listed in Supplementary Table 4.

### Phylogenetic Analysis of *qHTT8* Genes

The candidate genes for homologous genes in the other plants were obtained from the Rice Genome Annotation Project database (RGAP, http://rice.plantbiology.msu.edu/) database and the Phytozome (https://phytozome.jgi.doe.gov) database. Protein sequences of the candidate genes and their homologs from other species were retrieved from the Phytozome database. The phylogenetic tree was constructed using the Maximum Likelihood (ML) method with 1,000-replicate bootstrapping in MEGAX software (Kumar et al., 2018). An alignment of rice and its homologs from other species was performed using ClustalX v2.1. And the conserved domain of candidate gene was screened using the CD-search in the Nation Center for Biotechnology Information (NCBI, https://www.ncbi.nlm.nih.gov)database.

## Results

### Phenotypic Characterization of the HT of F_2:3_

**Figure 1:**
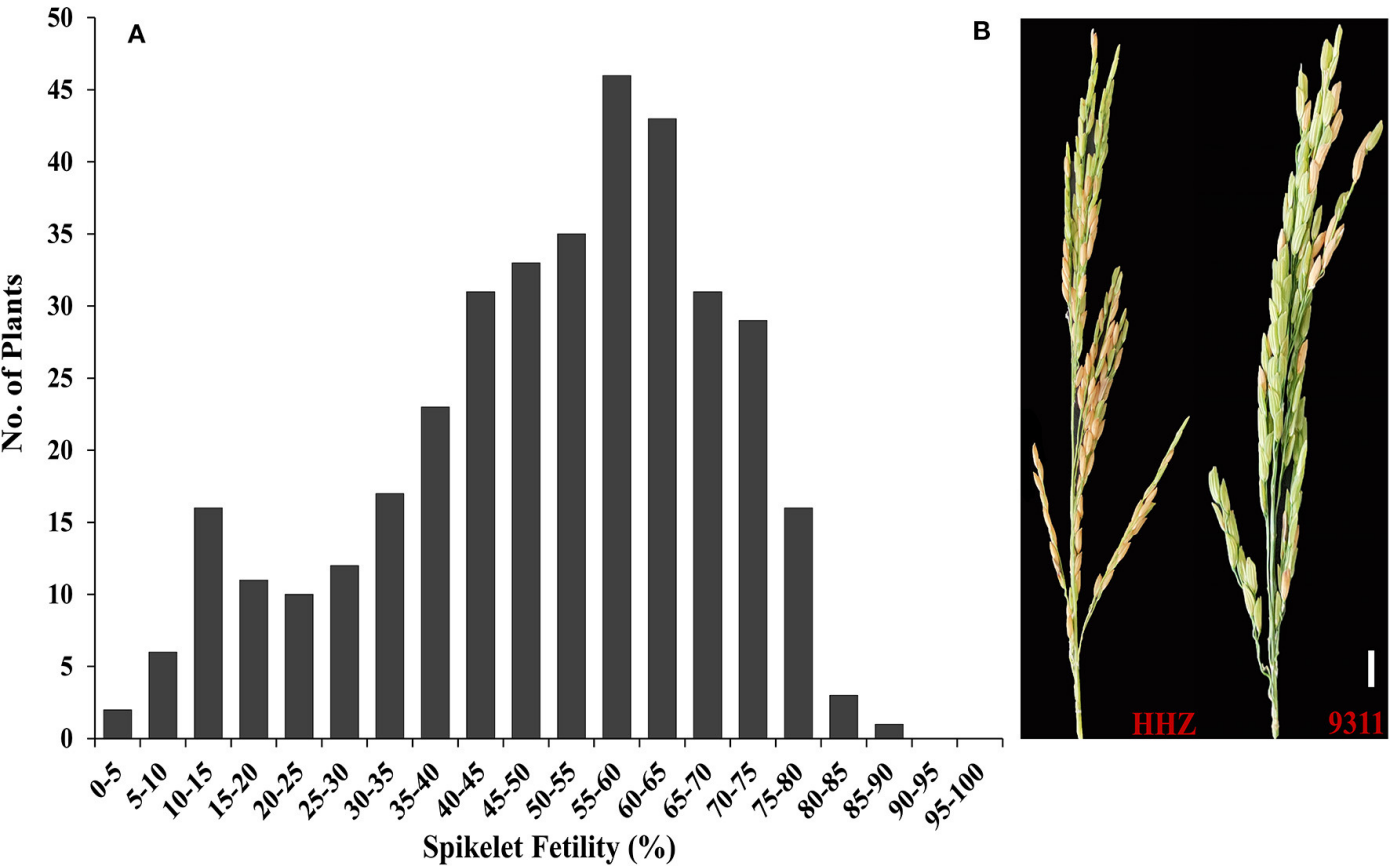
HT evaluation of the two parents and F_2:3_ population. **(A)** Frequency distribution of spikelet fertility in the F_2:3_ population containing 365 plants; **(B)** HHZ and 9311 under 38°C for 3 days.

Spikelet fertility has been previously used to screen and select for HT during the reproductive stage. To analyze the genetic basis of HT during the flowering stage in rice, we constructed an F_2:3_ population derived from crosses between HHZ and 9311. And the frequency distribution of spikelet fertility in the F_2:3_ population is shown in Figure 1A. Spikelet fertility ranged from 1.04 to 85.24%, with an average value of 50.00%, standard deviation of 0.1847 and coefficient of variation of 36.95%, indicating that the HT phenotype in the F_2:3_ population was normally distributed (Figure 1A). Meanwhile, the spikelet fertilities of their parents HHZ and 9311 were 54.5 and 14.3%, respectively, under high-temperature stress (38°C) for 3 consecutive days (Figure 1B). The F_2:3_ population showed a large degree of segregation and some super-parent lines because of their different genetic background, suggesting that the HT of rice at anthesis was a quantitative trait controlled by multiple QTLs/genes. To construct the heat-sensitive (S) and heat-tolerant (T) pools, 50 heat-sensitive and 50 heat-tolerant F_2:3_ plants were selected. The percentage of spikelet fertility of the 50 F_2:3_ plants in the HS-bulk ranged from 1.04 to 26.73% and that of the 50 F_2:3_ plants of the HT-bulk ranged from 69.90 to 85.24%.

### BSA-seq Analysis

Genomic DNA samples of the two parents (HHZ and 9311) and the two pools (T-pool and S-pool) were sequenced by an Illumina HiSeq^TM^ sequencer, and 85.7 Gb of clean data were generated after being filtered; the Q30 of all samples was >90% (Table 1), indicating the high quality of the sequencing data.

**Table 1:** The quality of sequencing data.

**Sample**	**Clean Reads (bp)**	**Clean Base (bp)**	**GC content (%)**	**Q30 (%)**
HHZ	55,685,598	16,765,143,297	43.77	94.13
9311	53,801,932	16,198,479,368	44.14	92.54
T-pool	86,378,436	26,006,038,749	44.14	93.85
S-pool	88,929,269	26,772,575,482	44.67	94.09

The parents and mixed-pool sequencing data were compared using Nipponbare as the reference genome, and mutations were detected (Table 2). The effective reads of HHZ accounted for 98.01% of the entire genome, with an average sequencing depth of 35.93×, whereas the effective reads of 9311 covered 98.14% of the entire genome with an average read depth of 35.30×. Both varieties showed good coverage and sequencing depth. BSA association analysis is a gene-mapping method based on mixed pool sequencing, which primarily analyzes regions with significant differences in the frequency of mixed pool genotypes to determine the QTL positions related to target traits. In this study, BSA was used to analyze the associated SNPs. Before association analysis, the SNPs were filtered to obtain 627,717 high-quality SNP loci. After filtering, 33,205 effective SNP loci with differences between the two pools were identified.

**Table 2:** Statistical analysis of sequencing depth and coverage.

**Sample**	**Mapped Ratio (%)**	**Properly Mapped (%)**	**Duplication Ratio (%)**	**Average Insert Size**	**Average Depth (×)**	**Real Depth (×)**	**Genome Coverage (1×) (%)**	**Genome Coverage (5×) (%)**
HHZ	98.01	90.51	18.44	378.7	35.93	38.52	93.26	89.5
9311	98.14	90.47	17.12	383.5	35.3	37.84	93.3	89.51
T-pool	97.8	90.23	17.87	384.5	56.01	58.5	95.75	92.63
S-pool	97.89	90.27	18.21	377	57.47	59.94	95.88	92.58

### QTL Mapping

**Figure 2:**
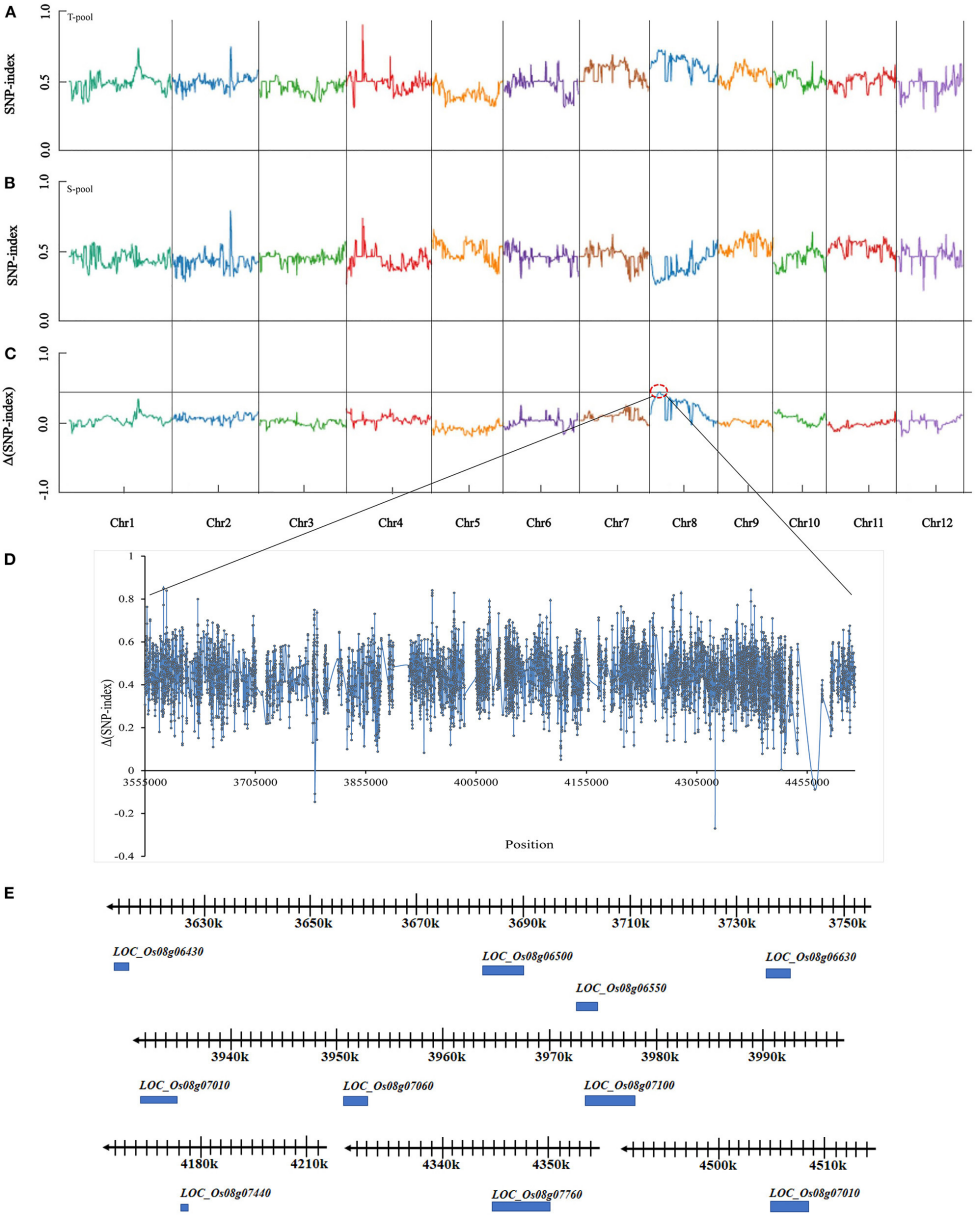
SNP-index graphs of T-pool **(A)**, S-pool **(B)**, and Δ(SNP-index) graph **(C)** from BSA-seq analysis. X-axis represents the position of the 12 chromosomes in rice; Y-axis represents the SNP-index. The black line shows the association threshold at a 99.9% confidence level **(C)**. HT major QTL is located to chromosome 8 (red dotted box). **(D)** Value of Δ(SNP-index) in the candidate region. **(E)** Physical location on chromosome 8 of the ten putative genes.

Based on resequencing and association analysis, the SNP-index of the T-pool and S-pool was compared (Figures 2A,B). At a 99.9% confidence level, the window above the threshold was considered the candidate interval. There was an imbalanced SNP between 3,555,000–4,520,000 bp on chromosome 8 (Figure 2C in the red-dotted box) (Figure 2D). In this region, the SNP-index value of the T-pool (heat-tolerant type) was greater than or equal to 0.7, while that of the S-pool (heat-sensitive type) was ≤0.3, indicating that the single plant in the heat-tolerant pool had the same fragment as HHZ in this region and that the single plant in the heat-sensitive pool had the same fragment as 9311 in this region. With a 99.9% confidence level as the screening threshold, the value of Δ(SNP-index) in this region was greater than the screening threshold. Therefore, the region of 3,555,000–4,520,000 bp on chromosome 8, which was named *qHTT8*, may be the putative locus controlling the HT of rice at the flowering stage. Analysis of the gene sequence of the *qHTT8* interval revealed a total of 6,821 SNPs and 1,155 InDels; 258 SNPs and 29 InDels caused amino acid changes.

### Gene Ontology (GO) Enrichment Analysis

**Figure 3:**
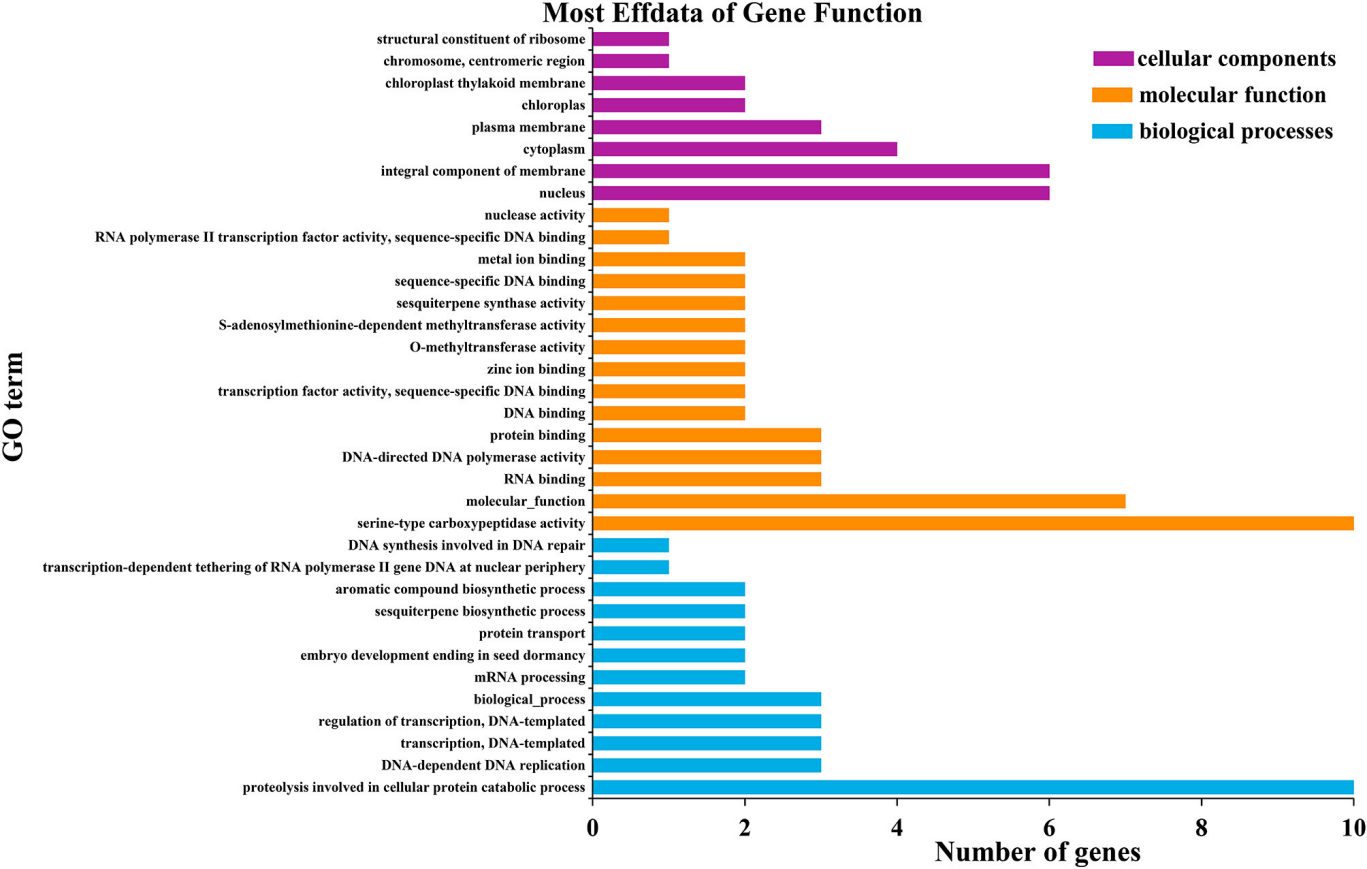
Significantly enriched GO terms of the genes around *qHTT8*.

Gene ontology (GO) analysis was used to classify all of the genes expressed into different functional categories, including biological processes, cellular components and molecular functions. Genes located in the genomic regions for the identified QTL were extracted (Supplementary Table 2). The *qHTT8* QTL harbored 53 genes that were annotated in the GO database. A total of 119 GO terms were grouped into the three categories, within which genes corresponding to biological process (46) and molecular function (39) were the most abundant. The proteolysis involved in the cellular protein catabolic process (GO:0051603) was the most significant in the biological process category, indicating that the rice leaves under heat treatment had wide metabolic activities. In the molecular function category and cellular component category, the serine-type carboxypeptidase activity (GO:0004185) and integral component of membrane (GO: 0016021) were the most significantly represented groups, respectively (Figure 3).

### Candidate Genes Analysis

To further analyze the candidate genes in the chromosome region containing *qHTT8*, we predicted 65 putative genes in the Nipponbare genome using the RGAP database (Supplementary Table 3). Ten genes that were located within the mapped region have been reported to be involved in abiotic stress tolerance, such as high night temperature, drought, cold, salinity and saline-alkaline (Table 3 and Figure 2E) (Ma et al., 2009; Smita et al., 2010; Nguyen et al., 2014; Lee et al., 2015; Raineri et al., 2015; Saha et al., 2015; Huang et al., 2017; Patil et al., 2017; Li N. et al., 2018; Hoang et al., 2019; Yang et al., 2019).

**Table 3:** Putative genes associated with abiotic stress tolerance in the *qHTT8* region.

**No**.	**Gene**	**Type and putative protein function**	**Physical location (bp)**
1	*LOC_Os08g06430*	Mitochondrial NADH-ubiquinone oxidoreductase, putative, expressed	3,613,350–3,615,878
2	*LOC_Os08g06500*	PPR repeat domain containing protein, putative, expressed	3,680,925–3,688,728
3	*LOC_Os08g06550*	Acyl CoA binding protein, putative, expressed	3,698,312–3,700,553
4	*LOC_Os08g06630*	RNA polymerase sigma factor, putative, expressed	3,732,441–3,736,521
5	*LOC_Os08g07010*	ABC-2 type transporter domain containing protein, expressed	3,928,462–3,933,577
6	*LOC_Os08g07060*	CRR6, putative, expressed	3,949,017–3,951,136
7	*LOC_Os08g07100*	Terpene synthase, putative, expressed	3,972,216–3,977,334
8	*LOC_Os08g07440*	AP2 domain containing protein, expressed	4,178,549–4,175,872
9	*LOC_Os08g07760*	BRASSINOSTEROID INSENSITIVE 1-associated receptor kinase 1 precursor, putative, expressed	4,344,171–4,350,502
10	*LOC_Os08g07970*	Transcription factor, putative, expressed	4,508,263–4,505,738

**Figure 4:**
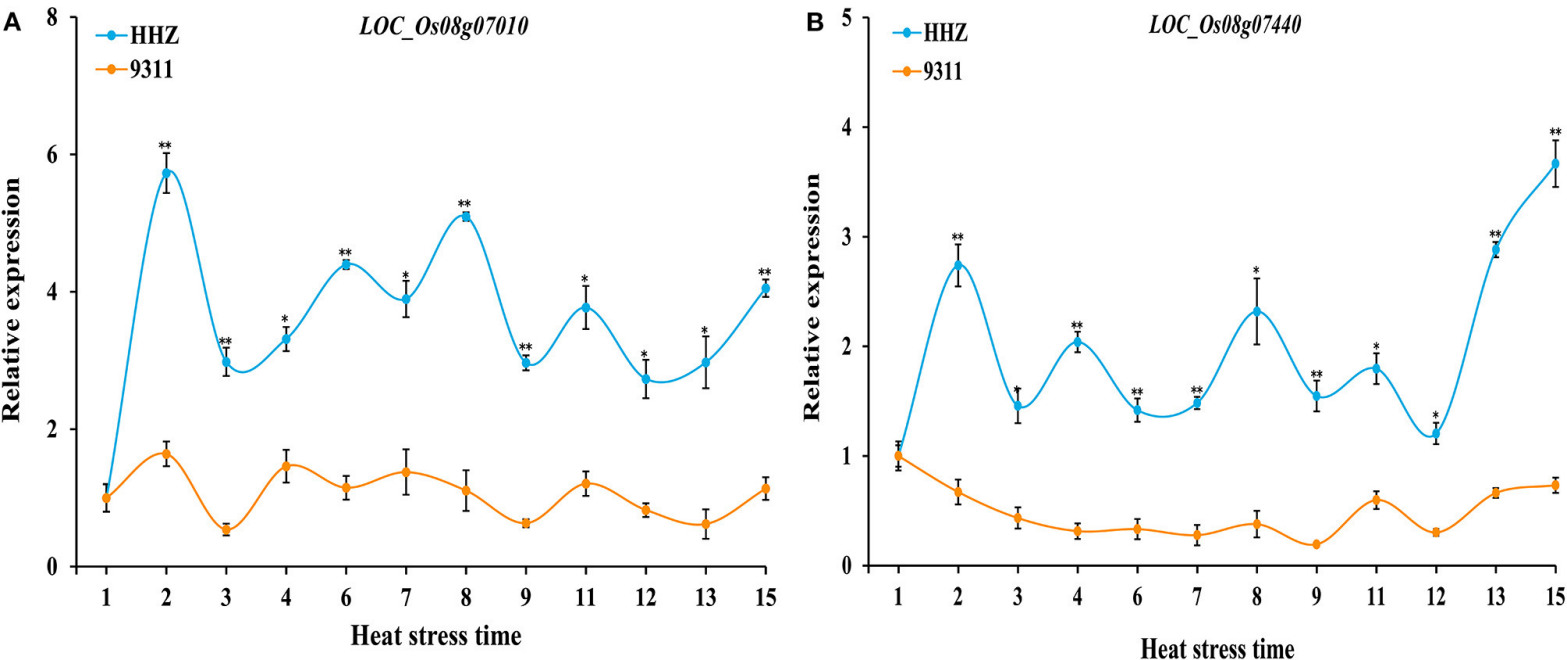
Expression analysis of candidate genes (**A**, *LOC_Os08g07010* and **B***, LOC_Os08g07440*) in HHZ and 9311 under heat stress for different time periods. The relative expression values were normalized to the rice *UBQ* gene. Error bars indicate standard deviation, and asterisks indicate significant differences using the Student's *t*-test (*p < 0.05; **p < 0.01). X-axis represents the different sampling time period for plants subjected to a 38°C heat stress in a phytotron; Y-axis represents the relative expression level of genes.

The relative expression levels of these 10 genes in the panicle between HHZ and 9311 were analyzed under different durations of heat stresses using qRT-PCR. Based on the cDNA sequences, 10 gene primers pairs for qRT-PCR analysis were designed. The qRT-PCR results showed that among these 10 genes, the expression levels of the genes *LOC_Os08g07010* and *LOC_Os08g07440* were both significantly higher in HHZ than in 9311 at all sampling points (Figure 4). In contrast, there was no clear differential expression between HHZ and 9311 in other genes (Supplementary Figure 1). Thus, *LOC_Os08g07010* and *LOC_Os08g07440* were determined to be the candidate genes responsible for the HT of rice.

### Phylogenic Analysis of *qHTT8*

**Figure 5:**
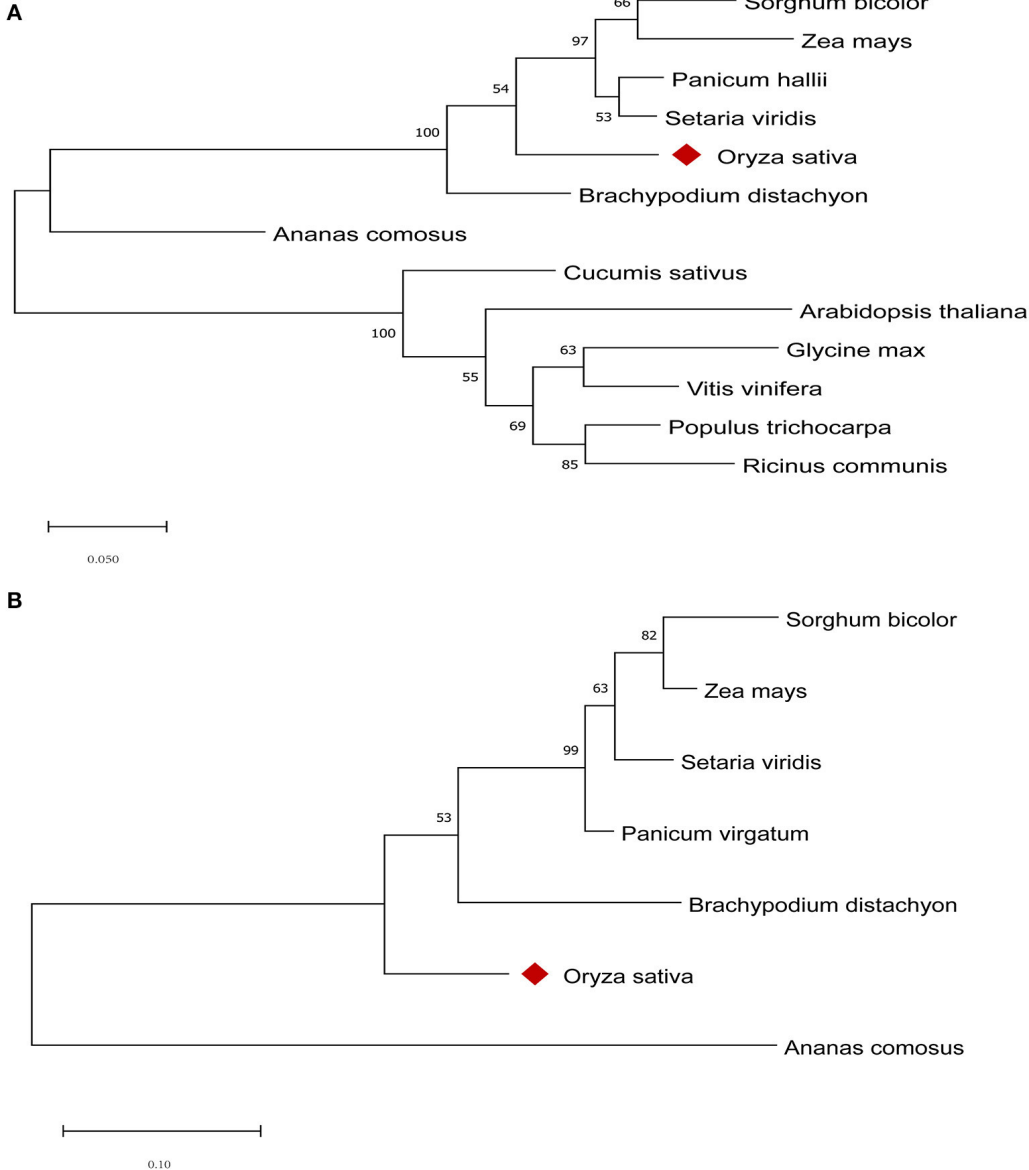
Phylogenetic tree of *LOC_Os08g07010* **(A)** and *LOC_Os08g07440* **(B)** homologs from different species. Phylogenetic analysis was carried out using MEGAX based on the ML method with 1,000 bootstrap replications.

**Figure 6:**
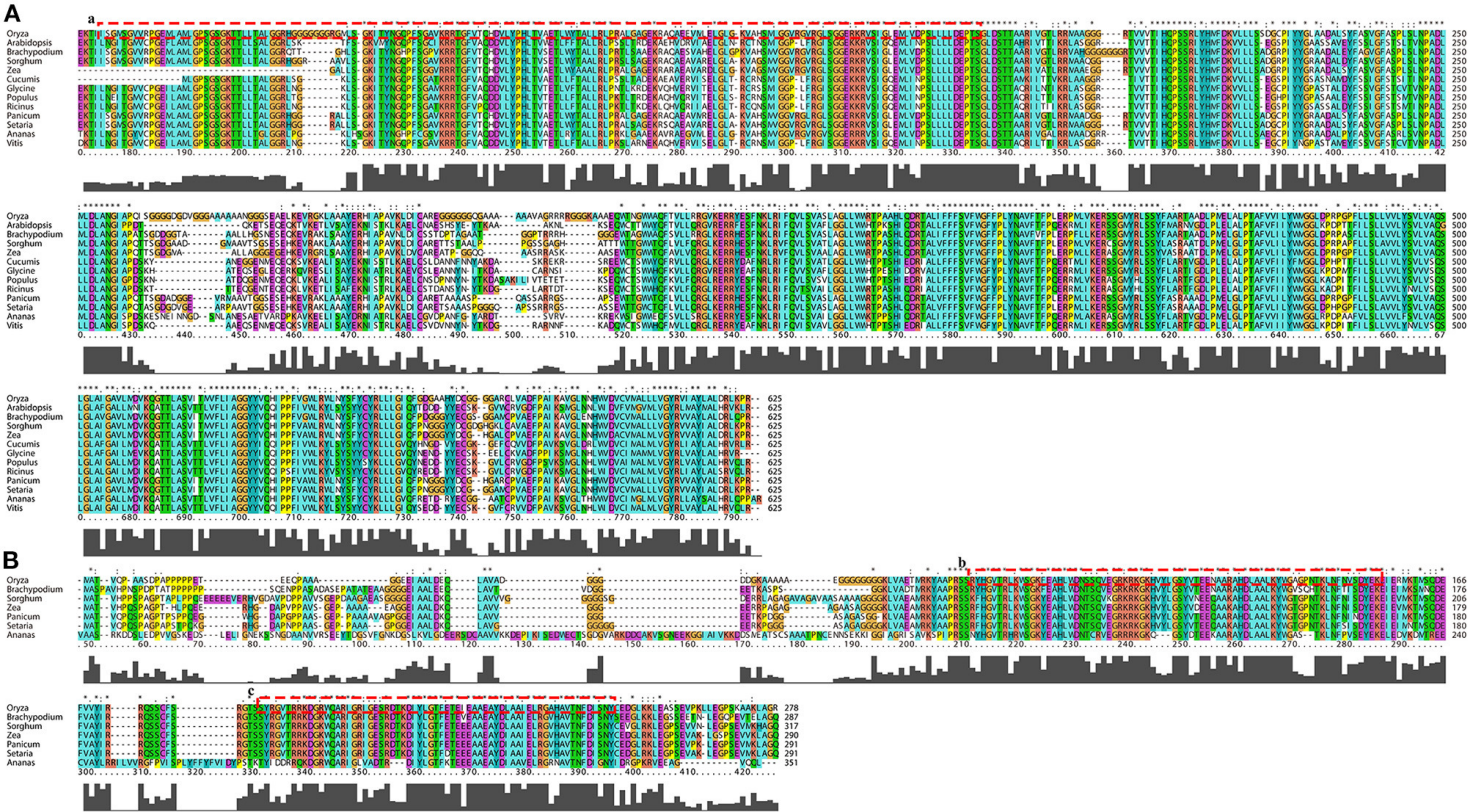
The amino acid sequence alignments of *LOC_Os08g07010* **(A)** and *LOC_Os08g07440* **(B)** homologs from different species. Amino acids marked with the same color in each column indicate 100% sequence identity. Gray columns with different heights represent the similarity of each amino acid sequence from different species. a, b, and c (the amino acid sequence inside the red dashed box) represent the conserved domains in *LOC_Os08g07010* and *LOC_Os08g07440*.

To better understand the similarities and differences in the two candidate genes in *qHTT8* between rice and other species, the phylogenetic tree was generated using different protein sequences from rice and other plants (Figure 5). *LOC_Os08g07010* (*OsABCG18*) encodes the ABC-2 type transporter protein. *AT1G31770* (*AtABCG14*) in *Arabidopsis thaliana*, one of the *LOC_Os08g07010* orthologous genes, was the first discovered protein related to the long-distance translocation of cytokinin which is one of the most critical signaling molecules in stress responses (Zhang et al., 2014). *OsABCG18* in rice and *AtABCG14* have similar biochemical functions in cytokinin long-distance transport from the root to the shoot (Zhao et al., 2019). Cytokinin plays an important role in plant growth, development and abiotic stress. *LOC_Os08g07440* encodes AP2 domain containing protein, which is a member of the AP2-EREBP family (APETALA2/Ethylene Responsive Element Binding Protein). AP2/EREBP genes also played a major and diversified role in plants to respond to various types of biotic and environmental stress (Riechmann and Meyerowitz, 1998; Dietz et al., 2010). *GRMZM2G022359* in maize, one of the *LOC_Os08g07440* orthologs, is involved in diverse abiotic stress responses and the regulation of processes (Huang et al., 2015). The phylogenetic analysis indicated that both *LOC_Os08g07010* and *LOC_Os08g07440* had high homology with most other homologous members, revealing that these two candidate genes were highly conserved. Furthermore, one and two conserved domains were found on *LOC_Os08g07010* and *LOC_Os08g07440*, respectively (Figures 6A,B).

## Discussion

With the threat of climate change, especially increased temperatures, droughts and desertification are expected to render several regions inhospitable to agriculture; consequently, the development of heat-tolerant rice cultivars is critically important for future rice production (Costa and Farrant, 2019). Over the past decades, researchers have identified dozens of QTLs controlling heat stress tolerance (Supplementary Table 1). However, as HT is a quantitative trait, its underlying genetic mechanism is relatively complex. To date, the use of these candidate genes to breed high-yielding, heat-tolerant rice varieties has been rare. There is thus an urgent need to conduct more research to identify candidate genes associated with the inheritance of rice HT.

In this study, we evaluated the HT phenotype of F_2:3_ families developed from HHZ crossed with 9311. The HT of F_2:3_ populations at the flowering stage was a quantitative genetic trait controlled by multiple QTLs/genes. BSA-seq combined with the conventional gene mapping method can significantly accelerate the fine mapping of genes (Zhang et al., 2020). The extreme expression of HT was selected via phenotypic identification to locate heat-tolerant QTLs at the anthesis of rice using BSA combined with WGS. A heat-tolerant QTL was located between 3,555,000 and 4,520,000 bp on chromosome 8.

Compared with previous studies, some QTLs/genes for HT have been identified on chromosome 8 in recent years. For example, Tabata detected a QTL for the occurrence of white-back kernels associated with high temperatures during the ripening period of rice at 0.15 Mb (Tabata et al., 2007). The QTL *qhr8-1* of HT at the flowering stage around 17.43–21.65 Mb was mapped by Cao, and *qtl_8.2* for absolute spikelet fertility near 20.53 Mb was detected by Jagadish, which overlapped the QTL *qHTGC8* for the thermo-tolerance of gel consistency in the 19.31–20.66 Mb region located by Cao et al. (2002); Zhu et al. (2006); Jagadish et al. (2010). The QTL *qht8* located by Chen in the interval of 5.59–39.4 Mb contained the *qhr8-1* located by Cao et al., the *qtl_8.3* (27.60 Mb) located by Jagadish, the heat-tolerant QTL ranging from 2,355,534 to 37,615,523 bp mapped by Zheng and the QTL of spikelet and pollen fertility (24.72 Mb) and the early morning flowering QTL of heat escape (22.34 Mb) mapped by Cao et al. (2002); Chen et al. (2008); Jagadish et al. (2010); Baliuag et al. (2015); Zheng et al. (2017). These QTLs above are different from *qHTT8* in physical location, therefore, they belong to the different loci. And the QTL *HD8* of days-to-heading (heat escape) in the range of 3.02–4.38 Mb detected by Thanh were located close to the QTL *qHTT8* (Thanh et al., 2010). However, due to the use of different evaluation index, the two loci are also different, thus, *qHTT8* represents a new QTL related to heat tolerance at the flowering stage in rice. Besides these, we did not find any QTL loci close to our interval on chromosome 8.

According to the RGAP database, 65 predicted genes were located in the target region containing *qHTT8*. Transcripts annotated as “hypothetical protein,” “expressed protein,” or “retrotransposon protein” were not included. The genes that were annotated to abiotic stress in rice (listed in Supplementary Table 3) were identified based on former studies. This analysis identified 10 annotated genes that were potential candidate genes for heat stress tolerance during the flowering stage in rice. Gene expression has been suggested to play the same role under different stress conditions. For example, *MYB*, a transcription factor (TF), was up-regulated when plants were exposed to a combination of drought and heat stress (Rizhsky et al., 2004). *OsbHLH148*, a basic helix-loop-helix TF, was responsive to heat, salt, dehydration and cold stress (Seo et al., 2011). *OsHCI1*, which is a rice gene encoding the RING finger protein, was specifically induced by heat and cold stress treatments but not by salinity or dehydration; its overexpression during heat and cold stress enhanced the acquired thermo-tolerance (Lim et al., 2013). Water deficits and high-temperature stress often occur simultaneously in the field (Bailey-Serres et al., 2019; El-Esawi and Alayafi, 2019; Shanmugavadivel et al., 2019). Furthermore, the expression of a trait might result from the contribution of many genes with similar or complementary functions (Ye et al., 2015a,b). These candidate genes, which play a role in other abiotic stress conditions, may thus have similar effects on the HT of rice at the flowering stage.

To confirm the candidate genes in *qHTT8*, we designed qRT-PCR primers to detect the expression level of the ten genes encoding proteins in HHZ and 9311 subjected to different durations of heat stress in a phytotron. Compared with other genes, the expression levels of *LOC_Os08g07010* and *LOC_Os08g07440* were significantly higher in HHZ than in 9311, indicating that they were highly induced by heat stress. Furthermore, phylogenetic analysis revealed that *LOC_Os08g07010* (*OsABCG18*) and *LOC_Os08g07440* were both highly homologous to genes from other species. *OsABCG18* and its ortholog *AtABCG14* were identified to play the same essential roles in transporting cytokinins from the root to the shoot. ABCG (ATP-binding cassette G) is one of the transporter families known to be involved in cytokinin transport in *Arabidopsis* and rice (Zhao et al., 2019). The overexpression of *AtABCG14* in *Arabidopsis thaliana* has been reported to improve drought resistance by regulating the inhibition of stomatal opening (Li, 2019). In our work, *LOC_Os08g07010* was found to be highly stress-inducible in a heat-tolerant variety, demonstrating that *LOC_Os08g07010* also plays an important role in the regulation of HT in rice, which may be related to the involvement of cytokinin in the heat stress response. In addition, the overexpression of *OsABCG18* (*LOC_Os08g07010*) was found to improve grain yield by increasing cytokinins in the shoot, further suggesting that this gene plays an important role in crop growth and development (Zhao et al., 2019). TFs play a central role in the response to abiotic stress. The AP2/EREBP superfamily, including four subfamilies (AP2, ERF, DREB and RAV), is one of the largest plant TFs (Riechmann and Meyerowitz, 1998; Muhammad et al., 2012). Previously, dozens of AP2/EREBP genes related to abiotic stress have been identified in plants, such as *OsDREB1A, HYR, OsEREBP1, OsERF48*, and *StDREB2* (Dubouzet et al., 2003; Ambavaram et al., 2014; Jisha et al., 2015; Jung et al., 2017; Mohamed and Aisha, 2019). Overexpression of these stress-related genes could improve abiotic stress tolerance, including tolerance of cold, heat, drought and salt stress. *LOC_Os08g07440*, a gene encoding the AP2 domain-containing protein, was highly induced by heat stress in HHZ than in 9311, suggesting that *LOC_Os08g07440* also plays a role in HT in rice.

In sum, *LOC_Os08g07010* and *LOC_Os08g07440* are two key genes controlling HT at the flowering stage in rice. However, how *qHTT8* affects the physiological changes of rice under heat stress and its molecular functions remain unclear. Additional study is needed to elucidate the role of *qHTT8* in the molecular mechanisms underlying the heat stress response during the flowering stage. Generally, the findings of our study have important practical implications for future efforts to improve the HT of rice.

## Conclusions

In conclusion, 6 h (9:30 am−3:30 pm) of exposure to high temperature (38 °C) for 3 continuous days is sufficient for identifying HT at the flowering stage in rice (*Oryza sativa* L.) (Liang et al., 2016). Ensuring that opened spikelets were removed before the plants were moved into the phytotron ensured the accuracy of the data. A new QTL for HT at the flowering stage in rice, *qHTT8*, was rapidly identified through BSA-seq within the region of 3,555,000–4,520,000 bp on chromosome 8. Ten putative genes controlling rice abiotic stress tolerance were also identified in this target region. Based on the qRT-PCR and sequence analysis, *LOC_Os08g07010* and *LOC_Os08g07440* were identified as the candidate genes controlling HT at the flowering stage in rice. This study provides a fast and effective strategy to identify heat-tolerant QTLs/genes at the flowering stage in rice. In future studies, functional analysis of parental lines will be used to validate the candidate genes by sequence analysis and genetic transformation.

## Data Availability Statement

The genetic sequencing data in our article have been deposited in the Nation Center for Biotechnology Information (NCBI) (SRA) database. The BioProject accession number is PRJNA674973, including four sub-accession numbers: SRR12998566, SRR12998567, SRR12998568 and SRR12998569 (https://www.ncbi.nlm.nih.gov/sra/PRJNA674973).

## Author Contributions

LJ and TL conceived and supervised the research. LC and QW designed the experiments. LC, QW, MT, XZ, YP, XY, GG, RL, and WT performed the experiments and analyzed the results. LC and QW wrote the paper. All authors have read and approved the final manuscript.

## Conflict of Interest

The authors declare that the research was conducted in the absence of any commercial or financial relationships that could be construed as a potential conflict of interest.
