# Supplementary figures and images for: QTL Mapping and Identification of Candidate Genes for Heat Tolerance at the Flowering Stage in Rice
Source: Front Genet. 2021 Jan 22;11:621871. doi: 10.3389/fgene.2020.621871 (PMC7862774; doi:10.3389/fgene.2020.621871)

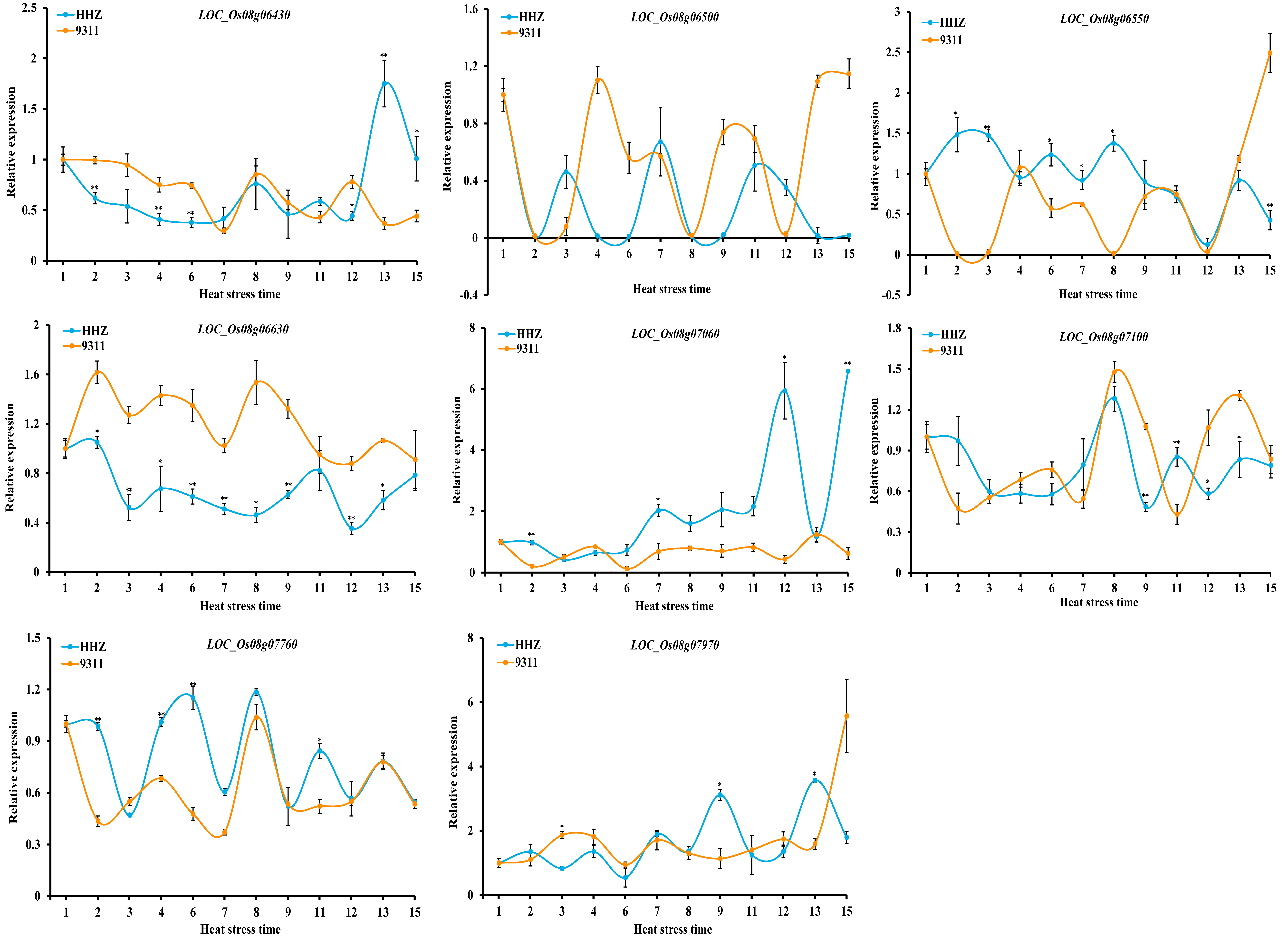

Supplement: Supplementary file 1 [file Image_1.JPEG]
